# Perception of King Khalid University students regarding the benefits, barriers, self-efficacy, knowledge, and practices related to healthy eating: a cross-sectional study

**DOI:** 10.7717/peerj.20523

**Published:** 2025-12-18

**Authors:** Amani Alhazmi, Manal Mohammed Hawash, Maha Ali, Haroon Ali, Farah Aziz, Khursheed Muzammil

**Affiliations:** 1 Public Health Department, College of Applied Medical Sciences, King Khalid University, Abha, Aseer, Saudi Arabia; 2 Department of Basic Medical Sciences, College of Applied Medical Sciences, King Khalid University, Abha, Asir, Saudi Arabia

**Keywords:** Healthy eating, Self-efficacy, Barriers, Nutrition, Knowledge, Beliefs, Practices, University students

## Abstract

**Background:**

Maintaining healthy eating habits is crucial for promoting better nutritional status, especially among university students who face challenges. Therefore, this study aimed to assess the perceptions of King Khalid University students regarding the benefits and barriers to healthy eating, their self-efficacy, nutritional knowledge, and dietary practices.

**Methods:**

This study employed a descriptive cross-sectional design. A total of 385 students participated by completing a pretested, self-administered questionnaire consisting of 87 items.

**Results:**

Findings revealed a high perception of the benefits of healthy eating (Mean = 4.23, SD = 0.83). However, participants reported significant perceived barriers (Mean = 3.12, SD = 1.19), particularly the high cost of healthy foods (Mean = 3.89, SD = 1.15). Self-efficacy in adopting healthy eating was moderate (Mean = 3.25, SD = 1.21). Knowledge of the health benefits of fruits and vegetables was high, whereas awareness of whole grains and flaxseeds was limited. Regression analysis indicated that perceived barriers (B = –0.181, *p* < 0.001), self-efficacy (B = 0.078, *p* = 0.003), and knowledge of food healthiness (B = 0.125, *p* < 0.001) were significant predictors of healthy food practices.

**Conclusion:**

Although students had positive perceptions, barriers and knowledge gaps persisted. Interventions should focus on reducing barriers, enhancing self-efficacy, and improving nutrition literacy to promote healthier behaviors among university students.

## Introduction

Healthy eating is vital for maintaining both overall physical and mental health, as it provides essential nutrients, supports various bodily functions, and helps prevent diseases such as obesity, thereby improving quality of life (QoL) (Almoraie et al., 2024; Cerqueira Sousa et al., 2022). The World Health Organization (WHO) emphasized that a balanced diet rich in nutrient-dense foods, while limiting processed foods, sugars, and unhealthy fats, significantly reduces premature death and improves QoL (World Health Organization, 2013). Promoting healthy dietary habits throughout life is an individual and societal responsibility, and respecting each region’s cultural heritage (Cena & Calder, 2020). Saudi Arabia’s rapid growth and urbanization have shifted diets from traditional foods to Western-style fast food, worsening dietary quality, raising the risks of overweight and obesity, and related health issues (Alhusseini et al., 2024; Alluhidan et al., 2022; Cena & Calder, 2020; Jalloun & Maneerattanasuporn, 2021) In Saudi Arabia, obesity is a major challenge, with a prevalence of 35% and an obesity-related death rate of 116.7 per 100,000 that far exceeds the global averages of 13% and 60 per 100,000, respectively (Salem et al., 2022). The problem is particularly significant among Saudi Arabia’s 1.7 million university students, a vulnerable population during this transitional phase of life (ICEF Monitor, 2025).

The transition to university marks a significant life stage that involves substantial lifestyle changes, especially in dietary habits, as students often struggle to maintain healthy diets when making independent food choices (Almoraie et al., 2024; Cerqueira Sousa et al., 2022; Yun, Ahmad & Quee, 2018). During this period, many students tend to skip meals, neglect breakfast, eat fewer fruits and vegetables, and rely more heavily on fried or fast foods, a pattern widely reported among European and Saudi university populations and linked to adverse health outcomes and weight gain (Alluhidan et al., 2022; Jalloun & Maneerattanasuporn, 2021; Yun, Ahmad & Quee, 2018). The university campus environment further exacerbates these unhealthy dietary choices through abundant fast-food outlets, vending machines, with limited healthy options alongside academic stressors, and sedentary lifestyles, which actively encourage reliance on ready-to-eat meals over nutritious home-cooked alternatives that may shape long-term health trajectories during this critical period (Almoraie et al., 2024; Cena & Calder, 2020; Yun, Ahmad & Quee, 2018). One recent Saudi research reported an alarming prevalence of overweight at 24.6% and obesity at 17.1% among Hail University students, linked to poor dietary and lifestyle patterns (Alrubaiee et al., 2025). This underscores the urgent need for Saudi Arabia’s Ministry of Health to implement targeted interventions, including university-based dietary guidelines and nutrition education programs aimed at promoting healthier eating among university students (Moradi-Lakeh et al., 2017). Nevertheless, the dietary behaviors and challenges faced by students at King Khalid University (KKU) in Saudi Arabia’s southern region remain underexplored. Understanding these dietary patterns is essential, as regional cultural and environmental influences may uniquely shape students’ eating habits. Therefore, investigating the factors influencing their dietary choices is vital to significantly improving their eating habits (Al Mulhem, El Alaoui & Pilotti, 2023; Cheikh Ismail et al., 2024).

In this context, nutritional knowledge is a key determinant of students’ dietary behaviors, as it is considered the foundation for making informed and healthy dietary choices. Assessing students’ understanding is crucial for gauging its impact on their eating habits and for designing effective university health promotion strategies (Almoraie et al., 2024; Cena & Calder, 2020; Moschonis, Magriplis & Zampelas, 2021). Research has consistently shown that higher nutritional knowledge significantly enhances university students’ ability to make healthier dietary choices (Ferrão et al., 2018; Guiné et al., 2023). However, another study found that while many undergraduates value a balanced diet, they often lack practical nutrition knowledge, such as understanding portion sizes and micronutrients, with the long-term effects of poor nutrition often persisting (Deshpande, Basil & Basil, 2009).

University students’ healthy dietary choices are also strongly influenced by perceived benefits and barriers, key constructs of the Health Belief Model (Miller & Steinle, 2020). These constructs are crucial for understanding how students weigh options when choosing healthy eating behaviors. Perceived benefits refer to an individual’s beliefs of positive outcomes of engaging in a behavior, such as increased energy and improved health through healthy eating. Conversely, perceived barriers represent the individual’s view of the obstacles that hinder behavior change (Rosenstock, 1974). University students face multiple barriers that hinder the adoption of healthy eating habits. These obstacles include the high cost of nutritious foods, limited time for meal preparation, and a lack of appealing healthy options, all exacerbated by social influences such as peer pressure and cultural food traditions (Almoraie et al., 2024; Jalloun & Maneerattanasuporn, 2021; Wongprawmas et al., 2022). European narrative review (2024) highlighted busy schedules, stress, limited healthy food access, social pressure, and unaffordable options as major obstacles to students maintaining healthys diets (Almoraie et al., 2024). Additionally, high food prices and poor food environment governance were also found to be significant barriers to healthy eating among Australian university students (Keat, Dharmayani & Mihrshahi, 2024) Furthermore, limited nutrition knowledge, combined with academic stress, further makes it even harder to adopt healthy dietary habits (Cena & Calder, 2020; Wongprawmas et al., 2022).

Importantly, a persistent gap exists between perceived benefits and barriers among university students regarding healthy eating, which helps explain why some students, even with sufficient knowledge, fail to translate that knowledge into healthy behaviors (Sogari et al., 2018). Research shows that university students recognize health benefits but undervalue them compared to perceived dietary barriers, a pattern observed across different cultural contexts, including North American, European, and Asian university populations (Deshpande, Basil & Basil, 2009; Sogari et al., 2018; Wongprawmas et al., 2022). In the Saudi context, cultural norms, gender roles, and campus food environments further influence their perceptions of barriers; however, high awareness of health issues, the perceived benefits are not strongly internalized among University students (Almoraie et al., 2024; Jalloun & Maneerattanasuporn, 2021). This gap between knowledge and behavior is partly explained by the concept of self-efficacy, which reflects a student’s confidence in their ability to select, prepare, and consume healthy foods under various circumstances (Cha et al., 2014; Cheikh Ismail et al., 2022; Deshpande, Basil & Basil, 2009). Bandura’s social theory suggests that individuals with high self-efficacy are more likely to adopt and maintain healthy eating habits, even when facing barriers, as it is crucial for encouraging behavioral change (Bandura, 1977; Cha et al., 2014). Research has shown that high self-efficacy is linked to healthier eating habits, whereas low self-efficacy is connected to dependence on convenient but unhealthy fast food options that can lead to disordered eating patterns. (Bouwman et al., 2020; Ebrahim et al., 2019). Notably, self-efficacy can be influenced by external factors such as the availability of cooking facilities and supportive environments that facilitate the practice of healthy behaviors (Al Mulhem, El Alaoui & Pilotti, 2023). Thus, addressing barriers, bridging knowledge gaps, and enhancing self-efficacy collectively constitute critical components in promoting healthy eating during university years. This intertwined perspective underscores the complexity of dietary behavior and the need for multifaceted interventions that account for psychosocial and environmental factors (Sogari et al., 2018). Despite dietary self-efficacy, a key predictor of nutrition behavior change, it remains understudied among Saudi students, particularly at KKU. Investigating self-efficacy in this unique context, characterized by gender-segregated dining and communal eating, can provide critical insights for developing culturally effective dietary education strategies (Al Mulhem, El Alaoui & Pilotti, 2023; Cheikh Ismail et al., 2022).

Unhealthy eating behaviors not addressed during university can persist in adulthood, potentially leading to long-term health consequences (Almoraie et al., 2024; Salem et al., 2022; Yun, Ahmad & Quee, 2018). While maintaining healthy dietary habits is crucial, there is a lack of research on how nutritional knowledge, self-efficacy, perceived benefits, and perceived barriers collectively influence students’ eating behaviors in southern Saudi Arabia. This region’s distinct cultural, environmental, and socioeconomic context may further influence dietary patterns, yet remains underexplored. To fill this gap, this study aimed to examine the dietary habits of KKU students through the theoretical frameworks of the Health Belief Model and Social Cognitive Theory. By integrating these models, this research identifies key factors that influence healthy food choices. It also helps develop targeted interventions to promote healthy dietary habits and mitigate diet-related disease risks in this population.

## Materials and Methods

### Study design and participants

This research adopted a descriptive cross-sectional design. It was conducted at KKU in the Abha district of the Asir region, Saudi Arabia, involving 61,708 students across various colleges and departments. Eligibility criteria require students to be at least 18 years old and have access to digital survey tools. Both male and female students were invited to ensure diversity. Participation was voluntary, with digital informed consent. A non-probability convenience sampling technique recruited 385 students, estimated using the Raosoft formula, targeting a total population of 61,708 students with a 0.05 precision level. This yielded a calculated sample size of 385 participants with a 5% margin of error and a 95% confidence level (Raosoft, 2004).

### Data collection measurements

This study employed a structured, self-administered questionnaire consisting of 87 items, adapted from the validated Benefits, Barriers, Self-Efficacy, and Knowledge Regarding Healthy Foods Survey scale developed by Pawlak & Colby (2009), which was selected for its alignment with the study’s objectives and comprehensive assessment of healthy eating constructs, including perceived benefits and barriers, self-efficacy, and nutritional knowledge (Pawlak & Colby, 2009). Formal permission was obtained from the author to use and adapt the instrument for Saudi university students. The original tool was designed to assess beliefs, barriers, and self-efficacy related to healthy eating and food purchasing behaviors. The internal consistency of the original scale and its subscales was assessed by Pawlak & Colby (2009), who reported Cronbach’s alpha coefficients ranging from 0.611 to 0.956, showing good reliability. The original scale demonstrated strong psychometric properties, including Cronbach’s alpha values of 0.956 for perceived benefits, 0.904 for perceived barriers, and 0.951, 0.611, and 0.879 for the three self-efficacy subscales (negative affective, positive social, and difficult/inconvenient). The scale also demonstrated high internal consistency for self-efficacy related to healthy food purchasing (α = 0.936). Validation included expert review, cognitive testing, and high internal consistency. The instrument has been used and applied across culturally diverse adult populations, confirming its applicability in cross-cultural contexts (Amaro et al., 2017; Bradette-Laplante et al., 2017; Garcia, Valencia & Amaro, 2021). All items were rated on a 5-point Likert scale consistent with the original tool, with higher scores indicating more substantial perceived benefits, perceived barriers, or self-efficacy toward healthy eating.

The questionnaire was distributed online using Google Forms in Arabic and divided into six sections. The first section comprises 14 questions designed to elicit information on general sociodemographic characteristics, lifestyle factors, and anthropometric measurements. Demographic and clinical characteristics data, including participants’ age, gender, social status, educational level, college categories, financial status, and presence of chronic diseases. A questionnaire also assessed lifestyle patterns, covering various aspects, including sleeping patterns, smoking habits, and physical exercise. Participants were asked to self-report their height and weight based on their available health records or personal knowledge. Body Mass Index (BMI) was then calculated using the standard formula: weight in kilograms divided by the square of height in meters (kg/m^2^). Based on the WHO criteria, participants were categorized as underweight (<18.5), normal weight (18.5–24.9), overweight (25.0–29.9), or obese (≥30) (World Health Organization, 2000). The second section assessed participants’ perceptions of healthy eating, examining perceived benefits and barriers to adopting a healthy diet. It included 11 statements to measure benefits, rated on a 5-point Likert scale from 1 (strongly disagree) to 5 (strongly agree). The perceived benefits scale in this study demonstrated excellent reliability, with a high Cronbach’s alpha (α = 0.92). Perceived barriers were assessed with 12 items that reflected obstacles such as food cost, preparation time, or peer influence. These items were rated on a five-point Likert scale from “strongly disagree” to “strongly agree.” Higher scores indicate greater perceived barriers. The perceived barriers scale used in this study demonstrated good reliability (α = 0.80). The third section consisted of 17 statements assessing self-efficacy for maintaining a healthy diet, divided into three subscales: seven assessing negative emotional contexts, three addressing positive social situations, and six evaluating challenges or inconveniences. Additional items assessed confidence in shopping for healthy food options, such as low-fat or low-sodium products and whole grains. Responses were rated on a scale from 1 (not at all confident) to 5 (very confident), and the study’s overall self-efficacy indicated good reliability (α = 0.80). The fourth section assessed participants’ ability to correctly identify healthy *vs* unhealthy food items, such as fruits, vegetables, whole grains, and red or processed meats. It used eleven items rated on a five-point scale from 1 (very unhealthy) to 5 (very healthy), with an option for “I do not know” scored as zero. The Food Health Knowledge Scale in this study demonstrated good reliability (α = 0.85), while the Disease-Prevention Knowledge Scale showed excellent reliability (α = 0.89). Additionally, eleven items measured participants’ nutritional knowledge regarding how certain foods and dietary supplements can help prevent chronic diseases, including heart disease, diabetes, and hypertension. These were rated on a five-point scale from 1 (does not prevent at all) to 5 (strongly prevents), with “I do not know” scored as 0. The Cronbach’s alpha for this part was 0.89, indicating high reliability. The fifth section included 11 items assessing healthy food practices, asking participants about their intake of whole grains, fruits, vegetables, meats, legumes, and nuts. Responses were on a four-point scale: “I eat some but not enough,” “My intake is about right,” “I should eat more,” or “I should eat less”, and this study’s healthy food purchasing or practices scale demonstrated good reliability (α = 0.82).

### Instruments validity

In the present study, items were culturally adapted for Saudi university students by substituting food examples relevant to local dietary habits and applying a rigorous forward–backward Arabic translation process to ensure semantic accuracy and cultural equivalence. A panel of five experts in community medicine, nutrition, and public health reviewed the scales for content validity, confirming their appropriateness, relevance, and clarity. Convergent validity was tested through confirmatory factor analysis (CFA), which verified that the measurement model accurately reflected the underlying constructs. A pilot study with 38 students was then conducted to assess face validity and clarity. The results indicated that the scales were comprehensible and required no modifications, and pilot data were excluded from the final analysis. Following the completion of the cultural adaptation and validation procedures, the finalized questionnaire was approved for use in the main study.

### Recruitment and data collection procedures

Upon approval from the relevant institutional authorities, the recruitment was conducted electronically through the university’s official communication system (Tawasl), which delivers to students’ institutional email accounts. Before beginning the questionnaire, participants viewed an information page outlining the study objectives, confidentiality assurances, and the voluntary nature of their participation. Electronic informed consent was required to proceed, and no personal identifiers were collected. Duplicate submissions were prevented using Google Forms single-response authentication. Responses were accepted until the predetermined sample size of 385 was reached, at which point the survey form was closed. Data collection was conducted over a three-month period, from January to March 2025.

### Ethical approval and participant consent

The study adhered to the Declaration of Helsinki and received approval from the Research Ethics Committee at King Khalid University (HAPO-06-B-001; Approval No. ECM#2024-3174). All participants provided informed consent through an online questionnaire, with a statement indicating that submitting their responses constituted their consent to participate.

### Data processing and analysis

The statistical analyses were conducted using IBM Corp.’s SPSS software, version 27. The collected data were organized and presented in tables, with categorical variables represented as numerical counts and percentages. Composite scores were calculated by averaging item responses for each construct (perceived benefits, perceived barriers, self-efficacy, nutrition knowledge, food healthiness knowledge, and healthy food practices). This scoring approach is consistent with the original validated (Pawlak & Colby, 2009) instrument and is standard in psychosocial measurement (Pawlak & Colby, 2009). Spearman’s rho correlation coefficient was employed to explore the relationships among demographics, lifestyle, BMI, perceived benefits, perceived barriers, self-efficacy, knowledge of food healthiness, and the role of healthy food in disease prevention. A multiple linear analysis model established the relationship between healthy food practices, perceived benefits, perceived barriers, self-efficacy in eating and purchasing healthy food, food healthiness knowledge, and its role in disease prevention. The internal consistency of the six scale measurements in the current study was assessed using Cronbach’s alpha coefficient, ranging from 0.80 to 0.92, indicating good reliability (Gliem & Gliem, 2003). This further justified the creation of composite variables, supporting the unidimensional representation of each construct through the use of mean and standard deviation values. Statistical significance was set at *p*-values ≤ 0.05.

## Results

Table 1 reveals an overview of the demographic and lifestyle characteristics of the study participants. Among the 385 participants, the majority were female (63.4%). Most participants were aged between 18 and 25 years (78.4%). The majority (44.2%) were pursuing a bachelor’s degree. A majority (69.6%) were enrolled in health-related colleges. The majority of participants (57.7%) had a normal BMI, and the least were obese (11.4%). The presence of chronic diseases was reported by 8.8% of participants. A significant majority (74.5%) engaged in physical activity.

**Table 1:** Demographic and lifestyle characteristics of King Khalid University students (*n* = 385).

Variable	Categories	Frequency	Percentage (%)
Gender	Male	141	36.6
	Female	244	63.4
Age	18–25	302	78.4
	26–35	67	17.4
	36–45	16	4.2
Marital status	Married	48	12.5
	Single	337	87.5
Educational level	Diploma	159	41.3
	Bachelor	170	44.2
	Post-graduate	56	14.5
College	Health college	268	69.6
	Non-health college	117	30.4
Income	Independent & financially sufficient	123	31.9
	Independent & financially insufficient	118	30.6
	Not financially independent	144	37.4
Living with	Alone	44	11.4
	With family	341	88.6
Place of residence	Rural	66	17.1
	Urban	319	82.9
BMI categories	Underweight	52	13.5
	Normal	222	57.7
	Overweight	67	17.4
	Obese	44	11.4
Presence of chronic diseases	Yes	34	8.8
	No	351	91.2
Smoking	Smoker	27	7.0
	Non-smoker	358	93.0
Physical activity	Yes	287	74.5
	No	98	25.5
Sleep patterns	<6 H	161	41.8
	>6 H	224	58.2

Table 2 depicts the participants’ perceived benefits and barriers to consuming healthy foods. The responses to the perceived benefits were measured on a Likert scale. The highest-rated benefit was “Healthy foods make me healthier and more vibrant,” and the lowest was eating healthy foods helps me lose weight effectively & eating healthy makes it easier for me to follow a balanced diet. The overall mean for perceived benefits was 4.23 (SD = 0.825). The most significant barrier reported was “Healthy foods are expensive compared to junk foods,” and the lowest was “Preparing healthy foods takes a long time”. The overall mean for perceived barriers was 3.12 (SD = 1.193).

**Table 2:** Perceived benefits and barriers to healthy food consumption.

Statements	Mean	SD
**Perceived benefits**		
Eating healthy food helps me feel better overall.	4.19	0.769
Eating healthy foods helps me care for my body and maintain my health.	4.31	0.772
Eating healthy foods helps me lose weight effectively.	4.08	0.937
Eating healthy food enhances my access to the necessary nutrients.	4.16	0.873
Healthy foods make me healthier and more vibrant	4.40	0.744
Eating healthy foods provides me with the necessary energy for my daily activities.	4.30	0.843
Eating healthy food helps maintain my appearance	4.34	0.761
Eating healthy foods helps cleanse my body of toxins	4.37	0.783
Eating healthy food aligns with the medical advice I get from my doctor or nurse.	4.15	0.874
Eating healthy makes it easier for me to follow a balanced diet.	4.08	0.867
Eating healthy foods, such as avoiding junk foods, helps me keep my digestive system healthy.	4.19	0.857
**Overall**	**4.23**	**0.825**
**Perceived barriers**		
Healthy foods are expensive compared to junk foods	3.89	1.154
The taste of healthy food is not delicious to me.	3.10	1.232
Preparing healthy food takes a long time.	2.80	1.179
Healthy foods do not contain enough sugar	3.01	1.112
Healthy foods do not contain enough salt.	2.85	1.082
Healthy foods contain very little fat.	3.25	1.111
I don’t have enough food to satisfy my appetite.	3.20	1.165
I do not know how to find healthy foods in stores easily.	2.95	1.281
I do not know how to make healthy foods properly.	3.17	1.264
My family does not like to eat healthy food.	2.83	1.304
My friends do not prefer to eat healthy food	3.32	1.241
**Overall**	**3.12**	**1.193**

Table 3 represents the participants’ self-efficacy regarding eating and purchasing healthy foods, with responses measured on a Likert scale. Self-efficacy in emotional contexts varied, with the highest mean reported when participants felt happy, and the lowest self-efficacy scores were observed when participants felt depressed. When purchasing healthy foods, participants demonstrated moderate self-efficacy in selecting whole grains or cereals; however, the overall mean self-efficacy score was 3.25 (SD = 1.205).

**Table 3:** Self-efficacy in eating and purchasing healthy foods.

	Mean	SD
**EATING** **How confident do you feel about eating healthy foods under each circumstance**		
When I am bored	3.07	1.066
When I feel frustrated	2.89	1.126
When I am worried	2.94	1.175
When I feel alone	3.08	1.235
When I feel angry	2.96	1.239
When I feel depressed	2.86	1.229
When I feel nervous	3.51	1.199
When I feel happy.	3.76	1.105
When I feel satisfied and in good condition	3.62	1.196
Eating at a restaurant with my best friends.	3.31	1.305
When unhealthy foods are only available, eating healthily becomes more difficult.	3.21	1.269
I find it hard to eat healthy meals when I bring them myself	2.73	1.293
Eating a healthy meal seems like a burden or annoying	2.80	1.274
When eating a healthy meal requires cooking, it feels uncomfortable or irritating.	3.17	1.314
Replacing unhealthy foods with healthy foods is a great effort.	3.43	1.277
**PURCHASING ** **When you are grocery shopping, how confident are you in your ability to:**		
Eating unhealthy foods is often easier than eating healthy foods.	3.56	1.211
Choose whole bread or cereals in the store.	3.57	1.171
Choose low-fat dairy products (such as low-fat yogurt).	3.35	1.205
Choose foods that are low in sodium.	3.23	1.180
Choose foods that are low in saturated fat.	3.27	1.147
Choose foods that are low in cholesterol	3.49	1.157
Choose foods rich in dietary fiber	3.52	1.166
Choose foods that are low or free of fats	3.51	1.171
**Overall**	**3.25**	**1.205**

Table 4 portrays the participants’ knowledge about the healthfulness of various food items, categorized into six response options. Among the food items perceived as the healthiest, green leafy vegetables, such as cabbage and lettuce, had the highest mean score, with 75.8% of participants rating them as “Very healthy.” Conversely, processed meats such as sausages and hot dogs were rated among the least healthy, with 29.9% of participants labeling them “Very unhealthy.” Overall, the results highlight a strong awareness of the health benefits of vegetables, fruits, and legumes, whereas processed meats and certain grains are viewed with skepticism or uncertainty.

**Table 4:** Knowledge of participants regarding the healthiness of selected foods.

Items	I do not know No (%)	Very unhealthy No (%)	Unhealthy No (%)	Neutral No (%)	Fairly healthy No (%)	Very healthy No (%)	Mean (SD)
Whole wheat bread	67 (17.4)	24 (6.2)	41 (10.6)	60 (15.6)	116 (30.1)	77 (20.0)	2.95 (1.74)
Whole wheat cereal	49 (12.7)	17 (4.4)	29 (7.5)	94 (24.4)	111 (28.8)	85 (22.1)	3.18 (1.59)
Oatmeal or Porridge	36 (9.4)	6 (1.6)	14 (3.6)	62 (16.1)	108 (28.1)	159 (41.3)	3.76 (1.52)
Flaxseed	55 (14.3)	7 (1.8)	11 (2.9)	54 (14.0)	103 (26.8)	155 (40.3)	3.58 (1.71)
Unsalted nuts (viz. peanuts, walnuts, pecans)	31 (8.1)	8 (2.1)	7 (1.8)	55 (14.3)	100 (26.0)	184 (47.8)	3.91 (1.47)
Fruits	14 (3.6)	7 (1.8)	5 (1.3)	47 (12.2)	67 (17.4)	245 (63.6)	4.29 (1.22)
Orange/yellow vegetables, viz. carrots, pumpkins	15 (3.9)	4 (1.0)	4 (1.0)	33 (8.6)	51 (13.2)	278 (72.2)	4.43 (1.18)
Green leafy vegetables, viz. cabbage, lettuce	13 (3.4)	6 (1.6)	3 (8.0)	35 (9.1)	36 (9.4)	292 (75.8)	4.47 (1.16)
Beans such as lima or red/kidney beans	34 (8.8)	7 (1.8)	7 (1.8)	47 (12.2)	59 (15.3)	231 (60.0)	4.03 (1.54)
Red meat, viz., sheep or beef	21 (5.5)	8 (2.1)	13 (3.4)	50 (13.0)	111 (28.8)	182 (47.3)	3.99 (1.34)
Processed meat (sausage, hot dog, *etc*.)	25 (6.5)	115 (29.9)	42 (10.9)	50 (13.0)	69 (17.9)	84 (21.8)	2.71 (1.69)

Table 5 illustrates participants’ knowledge of healthy food and supplements in disease prevention. Responses were categorized into six levels. The highest mean score was recorded for taking vitamins and minerals, with 14.8% of participants believing it “strongly prevents” diseases. However, the lowest mean score was recorded for eating less trans-fat. Overall, the results highlight a lack of awareness among participants regarding the disease-preventive benefits of healthy food and supplements.

**Table 5:** Healthy food practices among participants.

Which of the following, in your opinion, may help prevent chronic diseases such as heart disease, diabetes, and hypertension?	I do not know No (%)	Does not at all prevent No (%)	Not prevent No (%)	Neither No (%)	Prevent No (%)	Strongly prevent No (%)	Mean (S.D)
Eat more fruits.	77 (20.0)	87 (22.6)	131 (34.0)	0 (0.0)	90 (23.4)	0 (0.0)	1.84 (1.39)
Eating more vegetables	110 (28.6)	69 (17.9)	98 (25.5)	0 (0.0)	84 (21.8)	24 (6.2)	1.87 (1.66)
Eating more whole grains	114 (29.6)	69 (17.9)	112 (29.1)	0 (0.0)	77 (20.0)	13 (3.4)	1.73 (1.55)
Eating more fiber	96 (24.9)	73 (19.0)	141 (36.6)	0 (0.0)	75 (19.5)	0 (0.0)	1.70 (1.37)
Eating less total fat	85 (22.1)	67 (17.4)	160 (41.6)	0 (0.0)	73 (19.0)	0 (0.0)	1.76 (1.33)
Eating less saturated fat	102 (26.5)	65 (16.9)	155 (40.3)	0 (0.0)	63 (16.4)	0 (0.0)	1.63 (1.32)
Eating less trans fat	111 (28.8)	66 (17.1)	141 (36.6)	0 (0.0)	67 (17.4)	0 (0.0)	1.60 (1.37)
Eating less cholesterol	102 (26.5)	66 (17.1)	148 (38.4)	0 (0.0)	69 (17.9)	0 (0.0)	1.66 (1.35)
Eating less sugar	88 (22.9)	62 (16.1)	158 (41.0)	0 (0.0)	77 (20.0)	0 (0.0)	1.78 (1.35)
Eating less salt	94 (24.4)	69 (17.9)	152 (39.5)	0 (0.0)	70 (18.2)	0 (0.0)	1.70 (1.34)
Taking vitamins & mineral supplements	89 (23.1)	72 (18.7)	112 (29.1)	3 (0.8)	52 (13.5)	57 (14.8)	2.07 (1.73)

Table 6 demonstrates the participants’ self-reported consumption patterns of various healthy food items, categorized into four responses. Among the respondents, some reported that their intake was “about right.” Conversely, processed meat had the lowest mean score, with 21.6% of participants indicating that they “should eat less of it,” reflecting awareness of its potential health risks. Overall, these findings suggest that participants generally believe their consumption of vegetables and fruits is adequate. Additionally, there is a growing recognition of the need to limit consumption.

**Table 6:** Knowledge of healthy food and supplements in disease prevention.

How often do you eat any of the following foods?	I should eat less of it. No (%)	I eat some, but not enough No (%)	I should eat more of it No (%)	My intake is about right No (%)	Mean (S.D)
Whole wheat grain.	40 (10.4)	97 (25.2)	64 (16.6)	184 (47.8)	3.02 (1.07)
Oatmeal or oatmeal	27 (7.0)	103 (26.8)	89 (23.1)	166 (43.1)	3.02 (0.99)
Flaxseed	35 (9.1)	114 (29.6)	107 (27.8)	129 (33.5)	2.86 (0.99)
Unsalted nuts	32 (8.3)	96 (24.9)	113 (29.4)	144 (37.4)	2.96 (0.98)
Fruits	25 (6.5)	76 (19.7)	113 (29.4)	171 (44.4)	3.12 (0.94)
Orange/yellow vegetables	16 (4.2)	73 (19.0)	126 (32.7)	170 (44.2)	3.17 (0.88)
Green leafy vegetables	15 (3.9)	72 (18.7)	118 (30.6)	180 (46.8)	3.20 (0.88)
The legumes	31 (8.1)	98 (25.5)	98 (25.5)	158 (41.0)	2.99 (0.99)
Red meat	33 (8.6)	75 (19.5)	97 (25.2)	180 (46.8)	3.10 (1.00)
Processed meat	83 (21.6)	90 (23.4)	62 (16.1)	150 (39.0)	2.72 (1.19)

Table 7 shows that the multiple regression model was statistically highly significant (*p* < 0.001), indicating that the predictors jointly influence the perceived benefits and barriers of eating healthy foods. The model explained 13% of the variance in the dependent variable (R = 0.36, R^2^ = 0.128, adjusted R^2^ = 0.119). This provides a clearer understanding of which variables have a strong influence on dietary behavior and helps guide targeted interventions to promote healthier eating habits. The analysis revealed that perceived barriers, self-efficacy, and beliefs about the healthfulness of foods were highly significant predictors of healthy food practices. In contrast, perceived benefits and beliefs regarding dietary supplements did not show a significant association with the outcome variable. These findings suggest that interventions to promote healthy eating should focus on reducing perceived barriers, enhancing self-efficacy, and improving individuals’ understanding of food healthfulness.

**Table 7:** Multiple regression analysis of predictors of healthy food practices.

Independent variables	Unstandardized coefficients		Standardized coefficients	*t*-value	*p*-value	95.0% Confidence interval	
	B	Std. error	Beta			Lower bound	Upper bound
Constant	26.550	2.701	–	9.830	0.000**	21.240	31.861
Perceived benefits	−0.024	0.048	−0.027	−0.507	0.612	−0.119	0.070
Perceived barriers	−0.181	0.040	−0.224	−4.588	0.000**	−0.259	−0.104
Self-efficacy in eating and purchasing healthy foods	0.078	0.026	0.153	3.028	0.003**	0.027	0.129
Knowledge regarding the healthfulness of selected foods	0.125	0.028	0.238	4.389	0.000**	0.069	0.180
Knowledge regarding healthy food and supplements in preventing diseases	−0.040	0.027	−0.075	−1.470	0.142	−0.093	0.013

**Note:**

** *p* < 0.001 as statistically highly significant.

## Discussion

The findings of this research reveal the traits of the participants, as well as their perceived strengths and limitations in relation to healthy eating. The theoretical framework for dietary habits is complex, encompassing psychological constructs such as self-efficacy and emotional states, as well as environmental factors and societal beliefs about food. The interface between dietary habits and psychological constructs is a topic of much interest in health psychology.

Research suggests that adopting healthy eating habits is associated with improved mental and physical well-being (Ceylan, 2023; Mozaffarian, 2016). Boeing et al. (2012) reported that individuals with a positive view of healthy eating are more likely to practice mindful eating, which aligns with our findings that consuming healthy foods enhances energy and health. A moderate healthy food choice was observed in the present study when assessing the self-efficacy of consumption and purchasing healthy foods among participants. This level of self-efficacy plays a critical role in shaping dietary habits. According to Social Cognitive Theory, self-efficacy plays a significant role in an individual’s capacity to act under various circumstances or undergo behavioral changes (Schunk & DiBenedetto, 2020).

Considering the participants’ attitude towards healthy food, the results appear to be precisely consistent with this belief. Supplementation with vitamins and minerals reaffirms participants’ faith in their preventive ability compared to diet change. These results account for the intricacies behind public opinions regarding nutrition and health, which are likely to influence food selection and overall health status (Spronk et al., 2014). This implies the necessity for public health interventions that educate individuals on the importance of balanced eating habits and the specific functions of foods in relation to disease prevention.

This study reveals a negative relationship between physical activity and perceived benefit, which contrasts with the findings of Zhang et al. (2021), who found that physically active respondents have higher health literacy. Researchers have attributed external factors, including time availability, to discouraging participation in health-promoting behaviors (Wilcox et al., 2006).

Self-efficacy and healthy food beliefs are positively related to healthy eating behaviors and are thus considered predictors of dietary choices. Higher self-efficacy can help individuals achieve a higher-quality diet and make healthier food choices more frequently. Positive health beliefs about the healthiness of food were a significant predictor of increased fruit and vegetable consumption (Devirgiliis et al., 2024). Our findings are consistent with health behaviour interventions, which imply that people’s beliefs about their health risks and benefits are central to influencing their health behaviors (Gallagher et al., 2025). The results, perceived barriers towards healthy eating are a negative predictor, demarcating the impact on food choice; however, failure to adhere to a healthy eating regimen results from price, accessibility, and time constraints (Wansink & van Ittersum, 2013). Our results underscore the need for practical interventions, including the provision of healthy and affordable food, gender-specific strategies, targeted nutritional education, and focused programs to promote positive behavioral patterns. Further studies are needed to understand dietary patterns, emotional barriers, and the long-term impact of lifestyle interventions on student well-being. Overall, the findings highlight that the determinants of healthy eating behaviors are multifactorial.

A notable limitation of this study is the demographic profile of the sample, which was predominantly female, non-smokers, of normal weight, and from health-related colleges. This overrepresentation of specific characteristics limits the generalizability of the findings to the broader student population at King Khalid University and to Saudi Arabia as a whole. Additionally, the cross-sectional design prevents causal inferences, and the use of self-reported data may introduce reporting bias.

## Conclusion

This study highlights the students’ perceptions regarding the benefits, barriers, self-efficacy, knowledge, and practices related to healthy food. Participants demonstrated growing motivation toward healthy living and increasing awareness of the benefits of healthy eating. However, affordability and taste perception hindered their consumption of healthier foods. Favorable attitudes were observed toward whole grains, fruits, and vegetables, which were primarily driven by perceived health benefits rather than knowledge, suggesting a need for targeted educational interventions. Enhancing nutritional knowledge, increasing the availability of affordable healthy food, and providing cooking skills training can support informed dietary choices and promote long-term health among young adults. Future studies should aim to include more diverse and representative samples to enhance the generalizability and applicability of their findings. Longitudinal designs are recommended to explore causal relationships between knowledge, attitudes, and dietary behaviors. Incorporating objective measures alongside self-reported data can reduce reporting bias and strengthen the validity of the results.

## Supplemental Information

10.7717/peerj.20523/supp-1 Supplemental Information 1 Raw data. The dataset includes responses from King Khalid University students who completed an 87-item questionnaire assessing perceptions of healthy eating benefits and barriers, self-efficacy, nutritional knowledge, and dietary practices.

10.7717/peerj.20523/supp-2 Supplemental Information 2 Questionnaire.

## Additional Information and Declarations

### Data Availability

The following information was supplied regarding data availability:

The raw data used for analysis are available in Supplemental File.
