# Supplementary material for: Perception of King Khalid University students regarding the benefits, barriers, self-efficacy, knowledge, and practices related to healthy eating: a cross-sectional study
Source: PeerJ. 2025 Dec 18;13:e20523. doi: 10.7717/peerj.20523 (PMC12718526; doi:10.7717/peerj.20523)
Supplement: Supplemental Information 2 [file peerj-13-20523-s002.pdf]

**Perceptions, Benefits, Barriers, Self-Efficacy, and Knowledge Regarding Healthy Eating among University Students**

**Dear Student**

We kindly invite you to participate in this questionnaire, which aims to assess the perceptions of King Khalid University students regarding the benefits, barriers, self-efficacy, and knowledge about healthy eating. Please note that the information you provide will remain confidential and will be used solely for research purposes. Answering the questions in this questionnaire may take about 7 minutes. We want to emphasize that your participation is completely voluntary. You may withdraw from the study at any time. The data collected by this study will be used for research purposes only. By participating in this survey, you are deemed to have agreed to participate in the study, and we thank you in advance for your cooperation.

**Age:**

Under 18

18 – 25

26 – 30

Over 30

**Gender:**

- Male
- Female

**Education Level:**

- Diploma
- Bachelor’s degree
- Master’s degree
- Doctorate

**College:**

- Health-related
- Non-health-related

**Income:**
- Financially dependent

- Not financially independent and dependent on others

- Financially independent and sufficient

- Financially independent, but income is insufficient

**Housing:**
- Living alone

- Living with friends

- Living with family

**Place of Residence:**
- Village

- City

Height (in centimeters, using English numbers): (        )

Weight (in kilograms, using English numbers): (        )

**Do you have any chronic diseases (e.g., hypertension, diabetes, heart disease, kidney disease, etc.)?**
- No

- Yes

**Physical Activity:**
- I exercise regularly

- I exercise irregularly

- I do not exercise at all

- 1 to 3 times per week

**Hours of Sleep:**
- 6 hours or less

- More than 6 hours

**Benefits of Eating Healthy Foods**

Participants were asked: "How much do you agree or disagree with the following statements?"

Response options: 1=Strongly Disagree, 2=Disagree, 3=Neutral, 4=Agree, 5=Strongly Agree

Eating healthy foods would help me feel better.

Eating healthy foods would help me to take care of my body.

Eating healthy foods would help me lose weight.

Eating healthy foods would help me get more nutrients.

Eating healthy foods would help me be healthier.

Eating healthy foods would give me the energy I need.

Eating healthy foods would help me to look young.

Eating healthy foods would help to cleanse my body.

Eating healthy foods would be consistent with the advice of my doctor or nurse.

Eating healthy foods would help me to follow a Biblical diet.

Eating healthy foods would help to keep me regular (avoid constipation).

**Barriers to Eating Healthy Foods**

Participants were asked: "How much do you agree or disagree with the following statements?"

Response options: 1=Strongly Disagree, 2=Disagree, 3=Neutral, 4=Agree, 5=Strongly Agree

Healthy foods are too expensive.

Healthy foods do not taste good.

Healthy foods take too long to prepare.

Healthy foods are not sweet enough.

Healthy foods are not salty enough.

Healthy foods are too low in fat.

Healthy foods do not satisfy my cravings.

I do not know how to find healthy foods in a grocery store.

I do not know how to prepare healthy foods.

My children don’t like to eat healthy foods.

My friends don’t like to eat healthy foods.

My husband/wife doesn’t like to eat healthy foods.

**Self-Efficacy for Eating Healthy Foods**

Participants were asked: "How confident are you that you can eat healthy foods under the following situations?"

Response options: 1=Not at all Confident, 2=Not Confident, 3=Neutral, 4=Confident, 5=Very Confident

When I am bored.

When I am frustrated.

When I am stressed.

When I am lonely.

When I am angry.

When I am depressed.

When I am anxious.

When I am happy.

When I am feeling good.

While eating out at a restaurant with close friends.

When only unhealthy foods are readily available.

When I have to prepare healthy meals for myself.

When eating a healthy meal is just too much trouble.

When eating a healthy meal means I have to cook it.

When substituting a healthy for unhealthy food is a pain.

When eating an unhealthy food is more convenient.

**Self-Efficacy for Purchasing Healthy Foods**
Participants were asked: "How confident are you that you can perform the following behaviors?"

Response options: 1=Not at all Confident, 2=Not Confident, 3=Neutral, 4=Confident, 5=Very Confident

Select whole grain bread or cereal in a grocery store.

Select low fat dairy products (e.g., yogurt).

Select foods that are low in sodium.

Select foods that are low in saturated fat.

Select foods that are low in cholesterol.

Select foods high in dietary fiber.

Select foods that are low in or free of trans fats.

**Beliefs about the Healthfulness of Foods**
Participants were asked: "How healthy do you think each of the following foods is?"

Response options: 1=Very Unhealthy, 2=Somewhat Unhealthy, 3=Neutral, 4=Somewhat Healthy, 5=Very Healthy, 0=I don't know

Whole wheat bread

Whole wheat cereal

Oatmeal or rolled oats

Flax seeds/meal

Unsalted nuts (e.g., peanuts, pecans, walnuts)

Fruits

Orange/yellow vegetables (e.g., carrots, squash)

Leafy green vegetables (e.g., collards, lettuce)

Beans (e.g., lima beans, kidney beans)

Red meat (e.g., pork, beef)

Processed meats (e.g., sausages, hotdogs)

**استبانة عن التصورات والفوائد والمعوقات والكفاءة الذاتية والمعرفة المتعلقة بتناول الأطعمة الصحية لدى طلاب الجامعة**

عزيزي الطالب /عزيزتي الطالبة

ندعوكم للمشاركة في هذا الاستبيان الذي يهدف إلى تقييم تصورات طلاب جامعة الملك خالد فيما يتعلق بالفوائد والعوائق والكفاءة الذاتية والمعرفة بالأكل الصحي. يرجى ملاحظة أن المعلومات التي تقدمها ستظل سرية وسيتم استخدامها فقط لأغراض البحث. قد تستغرق الإجابة على الأسئلة الواردة في هذا الاستبيان حوالي 7 دقائق. نريد أن نؤكد أن مشاركتك طوعية تماما. يمكنك الانسحاب من الدراسة في أي وقت. سيتم استخدام البيانات التي تم جمعها من خلال هذه الدراسة لأغراض البحث فقط. من خلال المشاركة في هذا الاستطلاع ، تعتبر أنك قد وافقت على المشاركة في الدراسة ، ونشكرك مقدما على تعاونك.

**العمر:**

اقل من ١٨

١٨- ٢٥

٢٦-٣٠

اكبر من ٣٠

**الجنس:**

ذكر

انثى

**المرحلة الدراسية**

دبلوم

بكالوريوس

ماجستير

دكتوراه

**الكلية:**

صحية:

غير صحي

**الدخل**

-تعتمد على الاخرين مادياً

-غير مستقل مادياً ومعتمد على الأخرين

-مستقل ماديا ويكفي

-مستقل مادياً و لا يكفي

**السكن**

-بمفردي

-مع اصدقائي

-مع الأسرة

**مكان السكن**

-قرية

-مدينة

الطول بالسنتمتر) رقماً بالإنجليزية(   )

الوزن بالكيلوجرام) رقماً بالإنجليزية(   )

**هل تعاني من أمراض مزمنة مثل) الضغط، السكري، امراض القلب، أمراض الكلى ....الخ(**

-لا

-نعم

**النشاط البدني**

-امارس النشاط البدني بإنتظام

-امارس النشاط البدني بشكل غير منتظم

-لا أمارس اي نشاط بدني

-مرة إلى 3 مرات في الأسبوع

**ساعات النوم**

6 -ساعات فأقل

-اكثر من 6 ساعات

**إيجابيات الأطعمة الصحية**

يساعدني تناول الأطعمة الصحية على الشعور بتحسن بشكل عام.

يساعدني تناول الأطعمة الصحية في العناية بجسمي والمحافظة على صحتي.

يساعدني تناول الأطعمة الصحية في فقدان الوزن بشكل فعال.

يعزز تناول الأطعمة الصحية من حصولي على العناصر الغذائية الضرورية.

الأطعمة الصحية تجعلني أكثر صحة و حيوية

يوفر لي تناول الأطعمة الصحية الطاقة اللازمة لممارسة الأنشطة اليومية.

يساهم تناول الأطعمة الصحية في الحفاظ على مظهري

يساعد تناول الأطعمة الصحية في تنقية جسمي من السموم

يتماشى تناول الأطعمة الصحية مع النصائح الطبية التي أحصل عليها من طبيبي أو ممرضتي.

يسهل تناول الأطعمة الصحية عليَّ اتباع نظام غذائي متوازن.

يساعدني تناول الأطعمة الصحية في الحفاظ على صحة الجهاز الهضمي، مثل تجنب الإمساك.

الاستجابات

أعارض بشدة.

-أعارض.

-أوافق

-أوافق بشدة

-محايد

-**سلبيات الأطعمة الصحية**

تعتبر الأطعمة الصحية مكلفة مقارنةً بالأطعمة الأخرى

مذاق الأطعمة الصحية ليس لذيذًا بالنسبة لي.

تحضير الأطعمة الصحية يستغرق وقتاً طويلاً.

-الأطعمة الصحية لاتحتوي على كمية كافية من السكر

الأطعمة الصحية لا تحتوي على كمية كافية من الملح.

الأطعمة الصحية تحتوي على كميات قليلة جدًا من الدهون.

-الأطعمة الصحية لا تشبع شهيتي بشكل كافٍ.

لا أعرف كيف أجد الأطعمة الصحية في المتاجر بسهولة.

لا أعرف كيفية تحضير الأطعمة الصحية بطريقة صحيحة.

عائلتي لا يحبون تناول الأطعمة الصحية.

أصدقائي لا يفضلون تناول الأطعمة الصحية

-الاستجابات

أعارض

-أعارض بشدة

-أوافق

-أوافق بشدة

-محايد

**<u>بيانات تتعلق بالكفاءة الذاتية</u>**

تناول الطعام

مدى ثقتك في قدرتك على تناول الأطعمة الصحية في كل ظرف

مقياس المشاعر السلبية

عندما أشعر بالملل

-عندما أشعر بالإحباط

عندما أشعر بالقلق

-

عندما أشعر بالوحدة

عندما أشعر بالغضب

عندما أشعر بالاكتئاب

-عندما أشعر بالتوتر

الاستجابات

غير واثق على الإطلاق.

-غير واثق.

-لا أدري.

-واثق جداً

-واثق.

**مقياس المشاعر الإيجابية الاجتماعية**

عندما أشعر بالسعادة والفرح.

-عندما أشعر بالرضى وبحالة جيدة

-أثناء تناول الطعام في مطعم مع أصدقائي المقربين.

الاستجابات

غير واثق على الإطلاق.

-غير واثق.

-لا أدري.

-واثق جداً.

-واثق.

-**مقياس الصعوبة/الازعاج**

عندما تكون الأطعمة غير الصحية هي المتاحة فقط، يصبح تناول الطعام الصحي أكثر صعوبة.

أجد صعوبة في تناول الوجبات الصحية عندما احضرها بنفسي

-تناول وجبة صحية يبدو وكأنه عبء أو مزعج

عندما تطلب تناول وجبة صحية الطهي، أشعر بأنها غير مريحة أو مزعجة.

استبدال الأطعمة غير الصحية بأطعمة صحية يعتبر مجهودًا كبيرًا.

تناول الأطعمة غير الصحية غالبًا ما يكون أكثر سهولة من تناول الأطعمة الصحية.

الاستجابات

-غير واثق على الإطلاق.

-غير واثق.

-لا أدري.

-واثق جداً.

-واثق.

**<u>الشراء</u>**

**<u>عند التسوق، ما مدى ثقتك في قدرتك على:</u>**

اختيار خبز أو حبوب كاملة في المتجر.

اختيار منتجات الألبان قليلة الدسم) مثل زبادي قليل الدسم.(

اختيار أطعمة منخفضة في الصوديوم.

اختيار أطعمة منخفضة في الدهون المشبعة.

اختيار أطعمة منخفضة في الكوليسترول

اختيار أطعمة غنية بالألياف الغذائية

اختيار أطعمة منخفضة أو خالية من الدهون المتحولة

الاستجابات

-غير واثق على الإطلاق.

-غير واثق.

-لا أدري.

-واثق جداً.

-واثق.

**أسئلة حول صحة الأطعمة المختارة**

خبز القمح الكامل.

حبوب القمح الكامل

-الشوفان أو الشوفان المطحون.

بذور الكتان.

المكسرات غير المملحة) مثل الفول السوداني، الجوز، الجوز الأمريكي.(

-الفواكه

الخضروات البرتقالية/الصفراء مثل الجزر، القرع.

خضروات الورقية الخضراء مثل الكرنب، الخس.

البقوليات مثل الفاصوليا الحمراء

اللحوم الحمراء مثل لحم الغنم أو لحم البقر.

اللحوم المصنعة) السجق، الهوت دوغ، إلخ.(

الاستجابات

-صحي إلى حد ما.

-صحي جداً.

-غير صحي إلى حد ما.

-غير صحي جداً.

-لا أعرف.

-محايد

كم مرة تتناول أي من الأطعمة التالية؟

خبز القمح الكامل

حبوب القمح الكامل.

الشوفان أو الشوفان المطحون..

بذور الكتان

-أتناول بعضها ولكن ليس بما يكفي

الفواكه

الخضروات البرتقالية/الصفراء

الخضروات الورقية الخضراء..

البقوليات

اللحوم الحمراء

اللحوم المعالجة

الاستجابات

أتناول بعضها ولكن ليس بما يكفي.

-كمية تناولي مناسبة.

-يجب أن أتناول أقل منها

-يجب أن أتناول المزيد منها.

**ما الذي تعتقد أنه قد يساعدك في الوقاية من أمراض مثل أمراض القلب، السكري، أو ارتفاع ضغط الدم؟**

تناول المزيد من الفواكه.

-تناول المزيد من الخضروات.

تناول المزيد من الحبوب الكاملة

-تناول المزيد من الألياف

-تناول دهون أقل

-تناول دهون مشبعة أقل.

-تناول دهون متحولة أقل

-تناول كوليسترول أقل

تناول سكر أقل

-تناول ملح أقل.

تناول الفيتامينات والمكملات المعدنية

-الاستجابات

لا أدري.

-لا أعرف

-لا يمنع على الإطلاق.

-لا يمنع.

-يمنع بشدة.

-يمنع.

-غير صحي إلى حد ما.
